# Protein supplementation in strength and conditioning adepts: knowledge, dietary behavior and practice in Palermo, Italy

**DOI:** 10.1186/1550-2783-8-25

**Published:** 2011-12-29

**Authors:** Antonino Bianco, Caterina Mammina, Antonio Paoli, Marianna Bellafiore, Giuseppe Battaglia, Giovanni Caramazza, Antonio Palma, Monèm Jemni

**Affiliations:** 1 Department of Sports and Exercise Science (DISMOT), University of Palermo, via Eleonora Duse, 2, 90146, Palermo, Italy; 2 Department of Sciences for Health Promotion "G. D'Alessandro", University of Palermo, Palermo, Italy; 3 Department of Anatomy and Physiology "Human Physiology section", University of Padua, Italy; 4 Regional School of Sport, CONI Sicilia, Italy; 5 Department of life and sport science, University of Greenwich, UK

## Abstract

**Background:**

It is known that supplement use is a widespread and accepted practice by athletes and people who attend commercial gyms. Little is known about protein supplement amongst people undertaking strength training in commercial gyms in Italy when compared to the US.

**Objective:**

The purpose of this study was to examine the use of protein supplementation, alone or in association with other supplements, and dietary behavior amongst regular fitness center attendees in Palermo, Italy.

**Design:**

Resistance training information have been collected from 800 regular fitness center attendees for the initial analysis. A specific questionnaire was generated for the experimentation. Data were collected using a face-to-face interview method. Supplement users were then compared to the non users and analyzed using a one-way ANOVA, Kruskall-Wallis, chi-square test or exact test of Fisher when appropriate.

**Results:**

30.1% of the respondents use dietary supplements during their training as a believe it is the "way to gain muscles and strength". Whey protein shakes (50.0%) mixed with creatine and amino-acids (48.3%) were the most frequent choices amongst the users. A majority of the subjects (34.0%) appeared to rely on their gym instructors' advice for their intake; a lower proportion (13.0%) consulted physicians, while none of them consulted nutritionists. A high consumption of milk has been noticed in both users (67,7%) and non-users (52,8%); supplement non-users consumed significantly more snacks and bakery products than users per week (P < 0.001), while users consumed significantly more protein-rich foods (P < 0.01) with a particular preference for meat (48.0%).

**Conclusions:**

A considerable number of regular strength training adepts consume protein supplements mixed with other products (mainly creatine and amino-acids). Limited numbers consult "dietary specialists" and rely mainly on their instructors. We emphasize on the importance of the dissemination of scientifically based information about supplementation in this environment and the promotion of updated educational programs for the instructors.

## Introduction

Nutrition is traditionally perceived as a crucial component of physical fitness and performance. In the last few decades, the increasing understanding of human nutrition and its effects on the metabolism have led to a wiser management of the intake and the subsequent sport performance.

Global supplement use in athletes is estimated to range from 40% to 88% [1-5], with over 30.000 supplements being commercially-available in the United States (US) [3-5]. More than 3 million people in the US alone are using or have used ergogenic supplements [4-7] believing they may enhance their strength and physical performances. These are also widespread amongst athletes at high school and collegiate levels. However, evidence suggests that supplements might be beneficial only for small subgroups of people [7-11].

Some authors compared socio-demographic characteristics, like age, gender, education and income, between users and non users of mineral supplements and found significant age-related and education-related differences [12-14]. Other authors showed that intake of various micronutrients from natural foods was higher amongst supplement users compared to non-users; they have also indentified different food preferences between the two groups [15-18].

Supplements are consumed for a variety of reasons. Many exercise active individuals utilize supplements to build muscle, gain strength, prevent future disease or illness and improve performance in sport. Also, studies have shown that people have different opinions about the use of supplements [7-9,18-26]. This finding might be explained by different cultures, type of exercise training and type of dietary supplements. Kaufman et al. [27] found that older persons were more likely to take multivitamin and mineral supplements, while younger persons were more likely to take creatine. The choice of supplements depends also on the reason of the exercise program [20] and/or the type of sport [7]. It has been demonstrated that a significant number of consumers learn about supplements from unqualified sources rather than health professionals [20,21]. One of the aims of this study is to find out if the situation is similar in Palermo, Italy.

Although it is largely known that supplement use is a widespread and accepted practice by athletes and people who attend commercial gyms, with a large range of brands and products in the market [19,20], compared to the US only few studies have been carried out in Europe and other continents in this topic. In particular, we have no references about protein supplement amongst the adepts of strength training in gyms in Italy. Therefore, the purpose of this study was to examine the use of protein supplements, alone or in association with other intakes and also to identify the dietary behavior amongst people who want to *"build up muscles" *in regular commercial fitness' users in Palermo, Italy.

## Methods

### Participants

Permissions to conduct a survey were obtained from the managers of a representative number of six fitness centers located in the inner city and the suburbs of Palermo in 2009. The fitness centers have been identified using a database of CONI register (National Olympic Committee Register for Sport and Fitness Associations). Using the database of fitness centers, a number of 800 people (20% of the total number), have been randomly selected as potential participants.

Only fitness/gym attendees who were taking part in strength training courses have been selected. All gym/fitness users practicing aerobic activities (such as Aerobic, Spinning, Step, circuit training, endurance and cardiovascular programs, etc...) were excluded. On the basis of these inclusion/exclusion criteria, a total of 207 participants were retained for the investigation.

### Questionnaire procedure

In order to evaluate supplements use, dietary behavior and other related information, a 19-items questionnaire was developed based on previously published studies [20-24]. An informal pilot survey was preliminarily conducted among 27 customers of two fitness centers in order to identify issues of timing, wording or minor clarifications. The pilot-interviewed subjects had similar demographics and educational level to the target population.

The instrument examined the use of dietary supplements and their nutrient content (protein in association with other supplements), dietary behavior, reasons for use, education level and occupation. This latest was categorized as sedentary, standing, manual work and heavy manual work, according to the EPIC physical activity questionnaires criteria [22]. Easy definitions of the supplements were provided to the participants. Completion of the questionnaire implied respondent consent to participate in the study. According to the Italian regulations, ethical approval was not required for this study. The questionnaire was completed using the face-to-face interview method during four months by the same investigator. The surveyed population was split between supplement users and non users for comparison.

### Data Analysis

Data analysis was performed using EpiInfo software version 3.2 (CDC, Atlanta, GA, US) and Statistica version 8.0 software for Windows (Tulsa, OK, US). The descriptive analysis was performed by calculating the means, standard deviations and the frequencies. Differences were assessed by one-way ANOVA test, Kruskall-Wallis, chi-square test or exact test of Fisher when appropriate. The associations between the variables under examination were evaluated using contingency tables. Statistical significance was set at P values ≤ 0.05.

## Results

### Demographics

207 questionnaires were collected at the end of the survey period representing 80 females and 127 males. Table 1 summarizes the socio-demographic characteristics of the respondents. The average age of the surveyed subjects was 26.3 ± 9.1 yrs. Almost a quarter (23.7%) had attended eight years in the primary and secondary education and 21.3% had graduated from universities (≥ 13 years of education). The majority of the subjects were males (61.4%) and attended gym for one to five years (47.0%). Their job type was self categorized as sedentary (12.1%), requires standing (34.8%), manual work (27.1%) and heavy manual work (26.1%). The frequency of their strength training was one to two hours, three to five times per week.

**Table 1:** Demographic and lifestyle characteristics of participants, Palermo, Italy

	Subjects	
	***Number***	***Percentage***

Age (yr)		

< 18	23	11.1%

18-30	136	65.7%

> 30	48	23.2%

Mean (SD)	26,3 ± 9,1 yr	

		

Education (yr)		

≤5	2	1.0%

8	49	23.7%

13	112	54.1%

> 13	44	21.3%

		

Gender †		

Female	80	38.6%

Male	127	61.4%

		

Body mass index		

		

< 25 kg/m^2^	149	71.9%

25 ≤ 30 kg/m^2^	51	24.6%

≥ 30 kg/m^2^	7	3.5%

		

Activity at work		

		

Heavy manual work	54	26.1%

Manual work	56	27.1%

Standing	72	34.8%

Sedentary	25	12.1%

		

Recreational activity		

		

Yes	93	44.9%

No	114	55.1%

### Supplement use

Participants were asked to acknowledge the type and frequency of use of all the supplements they were consuming at the time of the survey. The majority of the subjects reported they didn't take any dietary supplement (69.9%). When data were compared by gender, men appeared to be more likely to use protein supplements than women (34.1% v 23.8% respectively; *P = 0.06*). The use of supplements was lasting 2.6 ± 3.3 years without reaching a significant difference between genders. Preferred types of supplements and protein packaging by frequency of use are described in Table 2. Whey protein shakes (50.0%) in association with creatine and amino acids (48.3%) up to seven times per week (24.2%) was the most frequently consumed supplement (Table 2).

**Table 2:** Frequency and type of supplements used among participants

	Subjects	
	***Number***	***Percentage***

Supplements use		

No	145	69.9%

Yes	62	30.1%

		

Users of supplement by gender		

Male	43	34.1%

Female	19	23.8%

		

Frequency of use		

1 time per wk	8	12.9%

2 times per wk	5	8.1%

3 times per wk	13	21.0%

4 times per wk	11	17.7%

5 times per wk	9	14.5%

6 times per wk	1	1.6%

7 times per wk	15	24.2%

		

Protein supplements		

Whey protein shakes	31	50.0%

Egg protein shakes	15	24.1%

Protein bars	12	19.3%

Protein Gel	1	1.6%

Protein shake blends	3	4.8%

		

Other supplements*		

Multivitamin/mineral	3	4.8%

Creatine - Amino acids	30	48.3%

Amino acids	16	25.9%

Multivitamin/mineral - Creatine - Amino acids	8	12.9%

Multivitamin/mineral - Amino acids	1	1.6%

Creatine	4	6.4%

*Other supplements in association with protein supplements.

### Source of information about use of supplements

**Figure 1:**
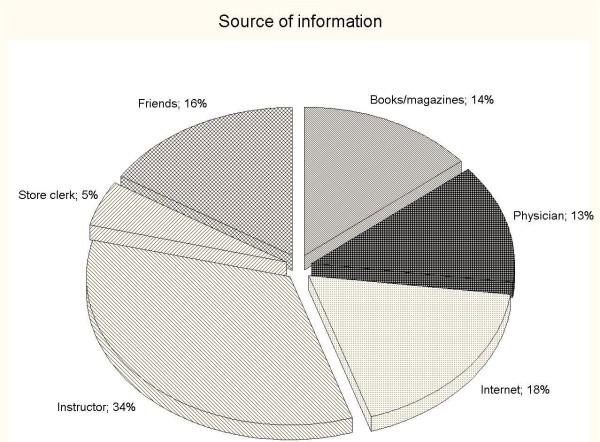
**Source of information about use of supplements**. Distribution of source of information amongst users.

When examining the source of information, a majority of the subjects (34.0%) appeared to rely on the gym instructors' guideline/advice, on the Internet (18.0%) or on "word to mouth" (16.0%). Only 13.0% of the participants consulted a physician, the shopkeepers at the stores were considered as a source of information by 5.0%. Unexpectedly, 14.0% of the participants used books or magazines as a source of information (Figure 1). Amongst the users, no one has consulted a nutritionist for advice on supplements.

### Dietary behavior

**Figure 2:**
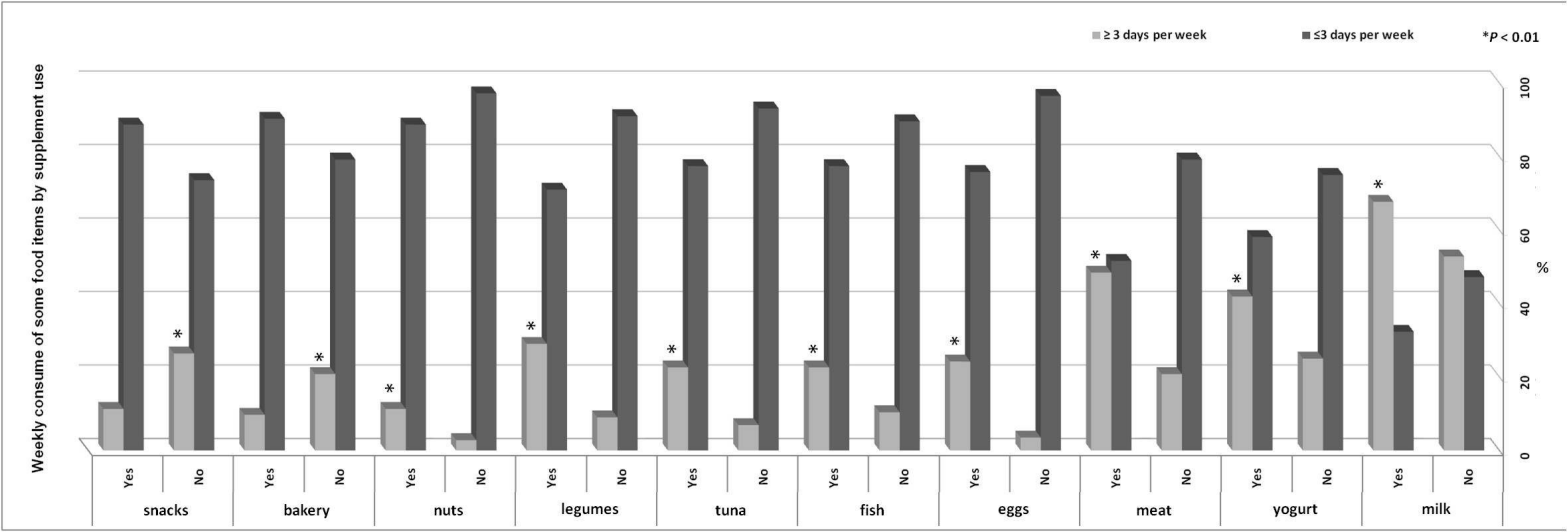
**Weekly consumption of some food items**. Weekly consumption of some food items by users (Yes) compared with non users (No), reported in ≥ 3 days per week and ≤ 3 days per week.

The survey showed that all groups consume milk more than three days per week [67% of the supplement users vs 52% in the non users (p > 0.05)]. However, the non-users consumed significantly more snacks and bakery products than the users per week (*P *< 0.001). On the contrary, supplement users consumed significantly more nuts, tuna, eggs, fish, legumes, meat, milk and yogurt than non-users (*P *< 0.01). The favorite high protein food of the both groups was meat (48.0%) (Figure 2).

## Discussion

Morrison et al. [20] compared supplement use by age group and found that young people consumed protein shakes/bars and creatine more than older people in the US. Other studies confirmed that the type of supplements used is age-related besides the type of exercise training [27-30]. Moreover, in Brazil, Goston and Correia [30] found that use of supplements was associated with the people who needed them less, since their diet appeared concurrently to be good or excellent. A similar observation has been described by Conner et al. [31] and Millen et al. [32]. Many authors suggest that athletes need extra protein in their diet as food or as supplements [33-37], however regular gym attendees do not need these extra supplements [30,34,37]. When comparing protein supplements by age and strength exercise training groups between our data and others from different studies, it appears that US has the highest prevalence of users with 59.8% among 85 subjects [20] followed by Brazil with 40.1% of users among 231 subjects [30]. Our survey showed 30.1% of supplement users amongst 207 subjects [Table 2]. According to other investigations, our study shows supplement consumption is more prevalent amongst men attending gyms [7,20,30]. Moreover, after stratifying protein supplements in whey protein shakes, egg protein shakes, protein bars, protein gel and protein shake blends, it appears that a majority of the users were taking whey protein shakes (50.0%), probably because they are thought to be the most effective. The questions that remain unanswered are: are they really more effective or rather more promoted by the media? And are they cheaper than others?

Our investigation also showed that younger supplement users did not habitually add multivitamin or minerals to their protein supplements. This finding is in accordance with previous studies [20,30].

In terms of source of information, we found that a high proportion of the subjects (34.0%) relied on the instructor. This was slightly lower than the rate found by Morrison et al. [20] amongst the American sample (38.7%), while Goston and Correia [30] reported only 14.1% of the users in Brazil relying on the gym instructors' guidelines. In this study, only few persons indicated consulting a physician for supplementation prescription (13.0%), a similar rate was reported by Goston and Correia [30] (14.6%), however, those rates were quite different to that reported by Morrison et al. [20]. In our sample of Italian fitness centers users, "word to mouth" was found to represent 16.0% of the information sources of supplementation, whilst Goston and Correia [30] reported 9.9% and Morrison et al. [20] 63.1%. It is important to underline that no one indicated consulting a nutritionist, whereas in Morrison et al' [20] and Goston and Correia' studies [30] the relative proportion is as high as 30.0%. It is clear that more studies are necessary to better understand this phenomenon.

In agreement with Goston and Correia [30], we found that users consumed more high protein food than non-users, in particular meat, but less snacks and bakery products than non-users. In addition, the use of supplements appears to be associated with persons who have already healthier dietary habits [38]. The sample size could be considered a limit of the study but considering strength and conditioning adepts only, most of the studies we found reported similar sample size [20,30]. This might be related to the difficulties to deal with managers and fitness adepts. In order to overcome these difficulties and to increase the sample a project named PP (Protein Project) is currently involving three European universities and the Italian National Olympic Committee (CONI). The results of this study will hopefully be published in future manuscripts and complete the current investigation.

## Conclusion

The percentage of supplement users was significantly lower in our study compared to others maybe because there is less marketing by protein supplement companies. This investigation showed a considerable number of adepts consumed protein dietary supplements in association with other high protein food. Whey protein shakes (50.0%) mixed with creatine and amino-acids (48.3%) were the most frequent choices amongst the users. The majority wasn't aware of the implications and the secondary effects of the supplements, since they were passively following their instructor's suggestions, got information from the Internet or from a friend. Only few obtained advice from a physician and none from a nutritionist.

As previously showed, we concluded that gym adept supplement users were not aware of objective recommendations for protein intake and may perceived their needs to be excessively high. It is generally accepted that athletes have increased protein needs. The position statement of the International Society of Sports Nutrition states that exercising individuals' protein needs are between 1.4 and 2.0 g/kg/day, depending upon mode and intensity of exercise, quality of protein, and status of total calorie and carbohydrate intake. General population attending commercial gyms usually had less workload than athletes, so daily protein intake should be in line with athletes guidelines or less.

Also, in agreement with previous studies, we think that it is extremely important to disseminate accurate information on the supplementation products mainly in the fitness centers. The promotion of updated educational programs and the integration of nutrition courses within the instructors' training will certainly help gym users achieving their objectives while guaranteeing less primary and secondary health risks.

## Competing interests

The authors declare that they have no competing interests.

## Authors' contributions

All authors have effectively contributed to this work in its different production stages. All authors read and approved the final manuscript.
